# Beyond the lab: future-proofing agriculture for climate resilience and stress management

**DOI:** 10.3389/fpls.2025.1565850

**Published:** 2025-06-13

**Authors:** Necla Pehlivan, Muhammad Tanveer Altaf, Abolghassem Emamverdian, Abazar Ghorbani

**Affiliations:** ^1^ Biology Department, Faculty of Arts & Sciences, Recep Tayyip Erdogan University, Rize, Türkiye; ^2^ Department of Field Crops, Faculty of Agriculture, Recep Tayyip Erdogan University, Rize, Türkiye; ^3^ Bamboo Research Institute, Nanjing Forestry University, Nanjing, China; ^4^ National Key Laboratory of Green Pesticide, Key Laboratory of Green Pesticide and Agricultural Bioengineering, Ministry of Education, Center for R&D of Fine Chemicals of Guizhou University, Guiyang, China

**Keywords:** sustainable agriculture, CRISPR, climate resilience, crop improvement, web of science

## Abstract

Biotechnology has revolutionized the agricultural landscape, ushering in a new era of crop improvement. Biotechnological delivery innovations have driven significant advancements, from enhancing nutritional value and shelf life to developing stress-resistant varieties. Leveraging techniques like genome editing, RNA interference (RNAi), and omics approaches, the potential to generate tolerant crops, create beneficial germplasm, achieve higher crop yields, and enable targeted biomolecule delivery has been unlocked, leading to the establishment of novel, sustainable agricultural systems. This review synthesized 481 studies sourced from the Web of Science (WOS) database, reflecting the diversity of plant species, biotechnological approaches, and abiotic/biotic stress categories reported in the literature over the past three years. The findings focused on specific applications and implications of various technologies across different stress categories and plant types, providing a detailed perspective on stress tolerance mechanisms. Furthermore, the review highlights significant areas of controversy, including ethical concerns, debates, challenges, risks, socioeconomic impacts, and limitations associated with these technological advancements. As the world’s population surges and dietary demands evolve, biotechnology holds the key to assuring secure food supplies and promoting sustainable agricultural practices amidst the challenges brought about by climate change. This synthesis highlights the significant potential of biotechnological advancements in revolutionizing agriculture, facilitating the creation of resilient, adaptable, and sound systems capable of addressing the needs of a swiftly evolving world.

## Graphical Abstract

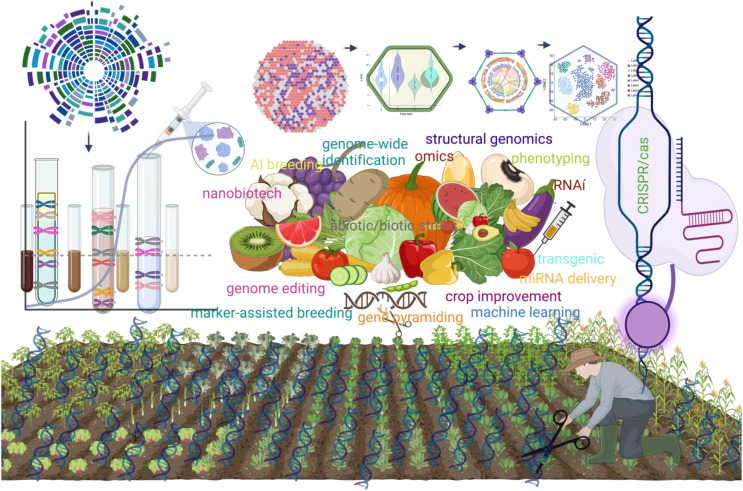

## Highlights

- Omics data is the highest (n=161), while CRISPR/Cas genome editing is trending among crops (n=45).
- Grain studies dominate, leaving fruits and vegetables underrepresented in abiotic stress research.
- Key species like *Oryza sativa* have often been used to improve stress tolerance over the last three years.
- Drought (n=94) in grains (n=236) was most prevalent, with the Australian pine invasion stress being the sole study screened.

## 1 Introduction

### 1.1 Revolutionizing agriculture in the current era

Humanity has previously relied on traditional agricultural practices for centuries to ensure its growing population (Turnbull et al., 2021; Prado et al., 2014; World Agriculture: Towards 2015/2030, 2023). While these methods laid the foundation for modern food systems, they often struggled to meet the needs of increasing populations and rapidly changing climates. In recent years, there has been a paradigm shift in agriculture as a result of the introduction of cutting-edge biotechnology instruments, which have revolutionized traditional farming processes and introduced unparalleled levels of efficiency. To illustrate, biomolecule delivery to plants has long relied on the traditional infection (with Agrobacterium) or biolistic particle delivery method. However, the research presented by Zhang et al. (2019) details a novel method for delivering biomolecules into plants by DNA nanostructures coordinating gene silencing in mature plants. The researchers successfully used nanostructures to transport siRNA into mature plant cells, effectively silencing targeted genes. Such technologies not only bypass traditional barriers but also enable broader applicability across diverse plant species. This versatility was further demonstrated in the development of maize varieties and rice lines, where genetic modifications in elite inbred lines, specifically altering granule-bound starch synthase 1(GBSS1), regulate amylose production. These adjustments resulted in a range of low amylose content of maize and rice lines (Zhu et al., 2020), with modified GBSS1, regulating amylose production. That is important because grain with less amylose has superior nutritional and appetizing qualities, along with extensive uses in the textile and adhesive industries. Other tecniques that are very different from traditional methods are robotics and artificial intelligence (AI) which are critical in enhancing crop stress tolerance using precision phenotyping, where robotic platforms and high-throughput imaging systems rapidly assess plant traits under stress (Mukherjee et al., 2025). It allows early stress detection using AI-driven sensors and drones that identify even subtle variations in plant health prior to any visible damage (Wang et al., 2025). These discoveries further optimize resource allocation through the implementation of smart irrigation systems that utilize AI to track water levels in the soil, climate, and plant needs, reducing unnecessary water loss for example. Besides, machine learning-based climate resilience models also allow breeders to develop crop varieties tolerant to future climatic stress factors (Das et al., 2025). In addition, these technologies accelerates breeding processes through the selection of stress-tolerant genes from vast genomic datasets, with automation optimizing CRISPR-based genome editing efficiency and accuracy (Bhuiyan et al., 2025). These outcomes highlight how targeted genetic modifications can simultaneously address dietary needs and industrial goals, further exemplifying the versatility of biotechnological interventions (e.g., gene-editing tools, synthetic biology, or soil microbiome engineering).

As the global population is projected to reach 9.7 billion by 2050 (United Nations, 2022) and as dietary shifts increase the demand for improved diets, there is a pressing need to significantly increase crop yields while sustainably producing foods (Lal, 2016). To address this challenge, a multifaceted approach will be crucial, one that leverages a combination of biotechnological innovations, advancements in traditional plant breeding, and the implementation of improved agronomic practices (Vemireddy, 2014; Husaini, 2022). The prospects for innovative solutions and tools adding up to the pile almost every year to drive significant advancements in crop improvement are indeed promising and multifaceted (Ranjha et al., 2022). The benefits of agribiotech are long-standing, with farmers worldwide embracing its diverse applications nowadays that span the spectrum of agricultural practices, including the development of better yielding varieties, enhancing nutritional profiles, and the production of biofertilizers. Innovations hold great potential to meet the increasing need for improved and sustainable crop production that is more tolerant to pressing agricultural issues, such as drought, high temperature, and soil salinity, which has started to occur more frequently and more dramatically.

The purpose of this study is to provide an assessment of current developments in biotechnology that have been made to increase crop resilience to environmental challenges. Modern molecular techniques, including CRISPR/Cas genome editing, marker-assisted breeding, and omics technologies, are the main focus here to improve crops. For instance, CRISPR/Cas genome editing for drought tolerance and other characteristics was outlined in the context of their implementation in applied crop breeding to create climate resilience. Similarly, omics technologies are not outlined as independent advances but as ways to interpret and improve crop adaptation to biotic and abiotic stresses. In addition to this, the review discusses the difficulties that are involved with the implementation of these technologies, such as ethical problems, regulatory impediments, and public acceptance. The purpose of this review is to suggest a road map for future research and policy development to promote sustainable agriculture practices and increase global food security. This is accomplished by summarizing the existing progress that has been made and identifying knowledge&policy gaps, and technological limitations.

### 1.2 Progress

#### 1.2.1 Drought tolerance

The sheer volume of the latest plant biotechnology data may render a comprehensive review impractical. However, recent experimental research on advancements in abiotic and biotic stress tolerance stands out, particularly the development of drought-tolerant crop varieties, which exemplifies the field’s rapid progress (Sharma et al., 2002; Vemireddy, 2014; Zhu, 2008). For example, genome editing and engineering techniques have been used to develop several drought tolerant crops (Raza et al., 2024; Rai et al., 2023; Ahmad, 2023; Sathee et al., 2022; Joshi et al., 2020) by modifying plant tissue-specific responses (Martignago et al., 2020). However, the basic strategy involves modifying genes that play key roles in response to drought stress, including overexpressing genes involved in drought signaling, such as those functional in the modulation of phytohormone signaling (e.g., abscisic acid signaling pathway) that enhance the ability to perceive and respond to drought conditions. Playing with signaling pathways has been shown in petunia and cassava plants as one of the examples (Estrada-Melo et al., 2015) (Zhao et al., 2014). Modifying genes that control root architecture and development via root system architecture (RSA) phenotyping was also among the biotechnological approaches for drought tolerance (Ndour et al., 2017) (Wajhat-Un-Nisa et al., 2023). In this context, genetic selections and the development of water deficit tolerant commercial maize varieties have been developed. This method can improve the plant’s capacity to access either water or nutrients from the soil during water scarcity periods. Manipulating genes responsible for the biosynthesis of osmoprotectant molecules that help plants maintain cellular integrity and function under water-deficit conditions are also among the valid methods used recently (Zhu et al., 2020; Zafar et al., 2020).

Beyond transgenic breeding with trans or cis-genesis (Jiang et al., 2017; Mishra et al., 2017; Pehlivan, 2019), precise genome editing by CRISPR has emerged as well, as a powerful tool to develop drought-tolerant crops by perfectly targeting specific genes in drought-specific stress responses (Jain, 2015; Esmaeili et al., 2022; Wang and Qin, 2017). CRISPR-Cas9 genome editing has enabled the targeted modification of genes to enhance drought tolerance in species such as *Arabidopsis*, rice (Kumar et al., 2023; Joshi et al., 2020; Paixão et al., 2019; Cao et al., 2017), wheat, maize (Rai et al., 2023), and several fruits, ornamental and industrial crops (Ramírez-Torres et al., 2021). Chronologically, molecular markers first have been essential in depicting plant genetic heterogeneity under environmental stressors. Numerous quantitative trait loci (QTLs) linked to enhancing drought resilience have been found in multiple crops (Puttamadanayaka et al., 2020). Gene mapping and germplasm evaluation enabled drought-tolerant wheat breeding, which can be categorized among climate-smart future crops (Khadka et al., 2020). Nonetheless, the accuracy and dependability of QTL identification seem problematic. In light of this, genome editing has proven highly effective in improving crop tolerance to abiotic and biotic stressors. Primary key genetic targets for this technique included those involved in ABA signaling, osmoprotectant synthesis, and root development (Sharma et al., 2002; Vemireddy, 2014; Zhu, 2008). However, there are novel sidesteps in this technology also: for instance, plant gene editing via *de novo* meristem induction is one of the novel techniques that bypasses the limitations of traditional tissue culture methods like delivering Cas9 and single guide RNAs reagents to explants in a culture for a typical plant gene-editing process. This old process was often genotype-dependent and could lead to unintended genetic changes. *De novo* induction, as described in (Maher et al., 2020), offers a more direct approach: 1. delivery: gene editing reagents and developmental regulator (DR)s are delivered directly to somatic cells of intact plants, often via Agrobacterium-mediated transformation. 2. the DRs induce the formation of new meristems at the site of delivery (meristem induction). 3. *de novo* meristems develop into shoots carrying the desired gene edits (shoot development). The key to this technique is the use of DRs, such as WUSCHEL and SHOOT MERISTEMLESS, which are essential for meristem formation and maintenance. The ectopic expression of these genes can trigger the emergence of new meristems in the target tissue. Simultaneous delivery of gene editing reagents (CRISPR-Cas9 components such as those needed for prime editing, adenine, and cytosine editing, or dual base editing) (Zhu et al., 2020) ensures that the newly formed meristems carry the desired genetic modifications. This method bypasses the need for lengthy and often problematic tissue culture procedures. Potentially higher efficiency and reduced time required for gene editing speed are gains compared to traditional methods. It also provides genotype independence and reduces surprise changes owing to its potential applicability to broader plant genotype groups and ability to minimize the risk of somaclonal variations associated with tissue culture. This work (Maher et al., 2020) successfully demonstrated the technique in *Nicotiana benthamiana* (a model tobacco) and *Solanum lycopersicum* (tomato). While further research is needed to optimize the method for other species and target tissues, the principle (efficient DRs delivery and editing reagents to target cells) remains crucial and still holds promise for a faster and more versatile approach for a wide range of dicotyledonous plants.

Furthermore, the application of nanotechnology has shown great potential in crop improvement to drought stress, with studies demonstrating the use of nano-scale materials to be able to have better water-use efficiency and photosynthetic activity in *Arabidopsis* and other major crops (Zhang et al., 2019; Kwak et al., 2019; Sharma et al., 2002; Vemireddy, 2014; Zhu, 2008). Emerging evidence suggests that chloroplast-selective gene delivery, together with nanoparticle-mediated approaches, can indeed enhance plant drought tolerance. Chloroplast-targeted genetic engineering has enabled the precise delivery and expression of involved genes in drought responses, such as those regulating ABA signaling, osmoprotectant synthesis, and root development, leading to more drought-tolerant crop varieties (Zhang et al., 2019). In this context, plant resilience to drought stress by enhancing water-use efficiency and photosynthetic activity in model species like *Arabidopsis*, as well as major crop plants, has been reported lately (Kwak et al., 2019). These advanced approaches, when integrated with traditional breeding and genome editing methods, offer significant potential for developing crops that are drought-tolerant and capable of surviving during climate change-related water shortages (Sharma et al., 2002; Vemireddy, 2014; Zhu, 2008).

#### 1.2.2 Thermotolerance

Recent studies have demonstrated omics-driven plant breeding for crops with higher tolerance to high and low temperatures (Demirer et al., 2019; Zafar et al., 2020). Systems biology approach and multi-omics data from integrated studies alongside machine learning algorithms and speedy breeding techniques blended of two or multiple omics methodologies within a singular study, conducted under identical or varying stress conditions and plant tissues, produced a thorough omics dataset, obtained mainly by the overexpression of heat shock proteins and the manipulation of cold acclimation genes for novel breeding programs aimed at creating temperature-smart cultivars (Zhou et al., 2022; Saeed et al., 2023). By leveraging these cutting-edge methods applied at tissue or single-cell levels (such as genomics, transcriptomics, proteomics, metabolomics, miRNAomics, epigenomics, phenomics, and ionomics) alongside the vast power of machine learning/speed-breeding data, essential stress-responsive genes and pathways have also been identified in plants to tolerate heat, cold, and salt stresses (Sezgin Muslu and Kadıoğlu, 2021). Machine learning analyzed the related data to evaluate plant thermotolerance responses and identified essential elements, including marker genes, metabolites, and proteins. Speed-breeding, on the other hand, expedited breeding cycles, enabling rapid introgression of desired traits and assessment of thermo-adaptive characteristics (Raza et al., 2024). Furthermore, electrophysiology and RNA-Seq of *Arabidopsis* STTM165/166 mutants revealed that Ca ion efflux in the cells was responsible for the generation of plant electrical signals regulating Ca^+2^ channel activity (Zhao et al., 2023). Also, through patch-clamp surface recording and differentially expressed gene ontology analyses, differential expression of electrogenic proton pumps (*Arabidopsis* H^+^-ATPases (also known as AHA genes)) generating slow wave membrane potentials has been shown to regulate plant electrical signals and temperature tolerance in this work.

#### 1.2.3 Salt tolerance

Innovations targeting other critical factors of stress, such as salinity, also signify substantial progress. The initial green revolution, fueled by chemical fertilizers, brought about a significant surge in food grain production; nevertheless, it also created a considerable issue: salinity (Gupta and Huang, 2014). When the ECe (conductivity of soil extract, saturated) is ≥ 4 dS/m, which is about the same as 40 mM NaCl and causes an osmoticum of 0.2 megapascal (MPa), the soil is considered to be salty in nature (Acosta-Motos et al., 2020; Corwin and Lesch, 2013; Ladeiro, 2012). In this context, even though the absence of a direct association between threshold salinity and yield reduction per unit rise in salinity, due to variations in salt exclusion, absorption, compartmentation, and other mechanisms of salt tolerance among various crop species, once posed a challenge, the genetic engineering of genes functioning in ion homeostasis, osmolyte biosynthesis, and antioxidant systems has enabled the development of tolerant plants that can grow and yield well in saline soils to date (Acet and Kadıoğlu, 2020; Fu and Yang, 2023). If we go back further, in 2000, Halfter et al. found that the SOS2 protein kinase physically interacts with and was activated by the calcium-binding protein SOS3, and emerging roles of the Salt-Overly Sensitive (SOS) pathway were established in *Arabidopsis* (Halfter et al., 2000). This knowledge paved the way for several metabolic manipulations: the overexpression of genes encoding sodium/proton antiporters, which regulate the Na^+^ efflux out of the cell, has been shown to improve tolerance in various crop species (Singh et al., 2021). Similarly, the engineering of genes involved in the biosynthesis of compatible solutes, such as glycine betaine and trehalose, helped plants maintain cellular osmotic balance under saline conditions (Deinlein et al., 2014). This was achieved by the classical transgenic approach (Pehlivan et al., 2016) to provide salinity tolerance to plants, which revolves around boosting internal defense mechanisms, often via a single-gene approach, sometimes gene pyramiding by a quantitative genetics process that is controlled by several numbers of genes simultaneously (Sun et al., 2018) On the other hand, in transcriptomic analysis, Zhao et al. (2022) found that stress-responsive transcription factors (TFs) interact with promoter areas to modulate the expression of salt stress-responsive genes associated with tolerance. Among grapevine TFs, the AP2-EREBP, bHLH, MYB, histone, WRKY, HSF, AuxIAA, and AS2 exhibited the most pronounced shifts (Zhao et al., 2022). Tolerant variants mobilized a greater number of bHLH, WRKY, and MYB TFs in response to salt stress compared to sensitive types. The manipulations in transgene research, which encode antioxidant enzyme genes such as SOD and CAT, enhanced the plants’ capacity to scavenge ROS and alleviated the oxidative damage linked to salt (Roy et al., 2014; Shams and Khadivi, 2023). Furthermore, the significance of halobiomes as a reservoir of genes for salt tolerance engineering in glycophytic crops was recently explored (Wani et al., 2020). Halobiomes, which consist of microbial communities adapted to thrive in the presence of high to very high salt habitats, serve as valuable reservoirs of stress-adaptive genes (Wani et al., 2020). These genes either functional for cellular Na^+^ influx or ion channel regulation, biosynthesis and transport of osmoprotectants, extracellular secretion, antioxidant machinery trigger etc. have potential applications in engineering salt tolerance in glycophytic crops (Chen S. et al., 2021), making halobiomes an important resource for agricultural biotechnology (Khare et al., 2024). The significance of microRNAs as essential post-transcriptional regulators in plant adaptive responses to salinity was also analyzed, along with a critical evaluation of their application to cultivating salt-tolerant crop plants such as grape, pepper, and alfalfa (Wei et al., 2023; Ma et al., 2022, 2020). Research on gene expression via constitutive promoters provided limited biological insights relative to the application of cell type-specific or inducible promoters. Consequently, the engineering of salt-tolerant plants achieved through miRNA overexpression, utilizing synthetic biology principles to improve engineering strategies, maintained homeostasis sor stable hormone levels to avoid pleiotropic effects, fully comprehended post-translational modifications, and precisely fine-tuned salt stress responses by engineering novel regulatory targets as reported by (Singh et al., 2021).

Recent research has investigated alternate splicing mechanisms and targeted techniques for gene editing to understand plant responses to salt stress further and to generate salt-tolerant crop cultivars (Ahmad et al., 2023). The several omics methodologies interconnected at the molecular level regarding salt stress tolerance in plant regulatory systems were illustrated alongside the roles of nano-biotechnology and microbiota (Xiao and Zhou, 2023). These improvements, when integrated with sophisticated breeding procedures and phenotyping technologies, have resulted in an array of salt-tolerant crops capable of surviving in regions with elevated soil salinity, hence enhancing the viability of agriculture and food security (Saradadevi et al., 2021; Singh et al., 2021; Afzal et al., 2022).

#### 1.2.4 Pest and disease resistance

Through the introduction of novel resistance genes, such as those encoding antimicrobial proteins, pathogen recognition receptors, and defense-related TFs or novel resistance genes from other organisms, such as Bt genes from *Bacillus thuringiensis* for insect resistance (Mapuranga et al., 2022) and the RFO (resistance to *Fusarium oxysporum*) genes from radish for fungal resistance, has also been a significant focus of biotechnology research and has led to the commercialization of several genetically modified (GM) crops (Chinnusamy et al., 2005; Roy et al., 2014; Hanin et al., 2016; Hernández, 2019). Endophytic microorganisms significantly contribute to host plant tolerance by eliciting systemic resistance, synthesizing more beneficial secondary metabolites, and aiding bioremediation (Koltun et al., 2018; Kaul et al., 2020; Bao et al., 2019). This has been a central focus of research confined to cultivating crops with enhanced disease resistance to biotic pressures attributed to genetic characteristics associated with stripe rust, leaf rust, stem rust, powdery mildew, fusarium head blight, and some insect pests. RNAi-based elements that give resistance to viral diseases were also essential components of varied integrated pest management tactics for various crops in countries with developed as well as developing economies (Li et al., 2022). After the innovation of the CRISPR system, the approach has been used to enhance resistance to late blight disease in potatoes by disrupting the susceptibility gene *StDMR6–1* and to confer resistance to powdery mildew in wheat by editing the mildew resistance locus A gene (Taj et al., 2022; Schenke and Cai, 2020). In contrast to traditional transgenic approaches that incorporate foreign herbicide-resistant genes (e.g., bar), which encodes phosphinothricin N-acetyltransferase into crops, the editing of herbicide-targeted genes to confer defense through CRISPR-Cas has more potential due to its rapidity, adaptability, and absence of transgenes. This is also significant as weed issues are escalating at a global scale; creating herbicide-resistant germplasms is a cost-effective way to sustain high crop yield and avert soil degradation in terms of habitat protection (Zhu et al., 2020). CRISPR-Cas9 has facilitated the development of herbicide-resistant varieties in several crops, allowing for more effective weed control (Zhu et al., 2020). The technology has been employed to enhance resistance against citrus canker disease and enabled the obtaining of wheat varieties resistant to powdery mildew disease (Zhu et al., 2020). The predominant method in functional genomics had previously emphasized the deletion via non-homologous end-joining repair of susceptibility elements essential for effective host colonization in plants (McGaughey and Whalon, 2023). However, the latest data indicated that genome re-engineering through homology-directed repair or base editing can inhibit host manipulation by altering the targets of pathogen-derived molecules (e.g., effectors) to the point of unrecognizability, hence reducing plant sensitivity (Veley et al., 2021). Due to these disadvantages, CRISPR/Cas genome editing became increasingly essential for quickly generating adaptive resistance characteristics in crops to address forthcoming challenges (Zhu et al., 2020).

#### 1.2.5 Enhancing nutrients/nutrient use efficiency

**Figure 1:**
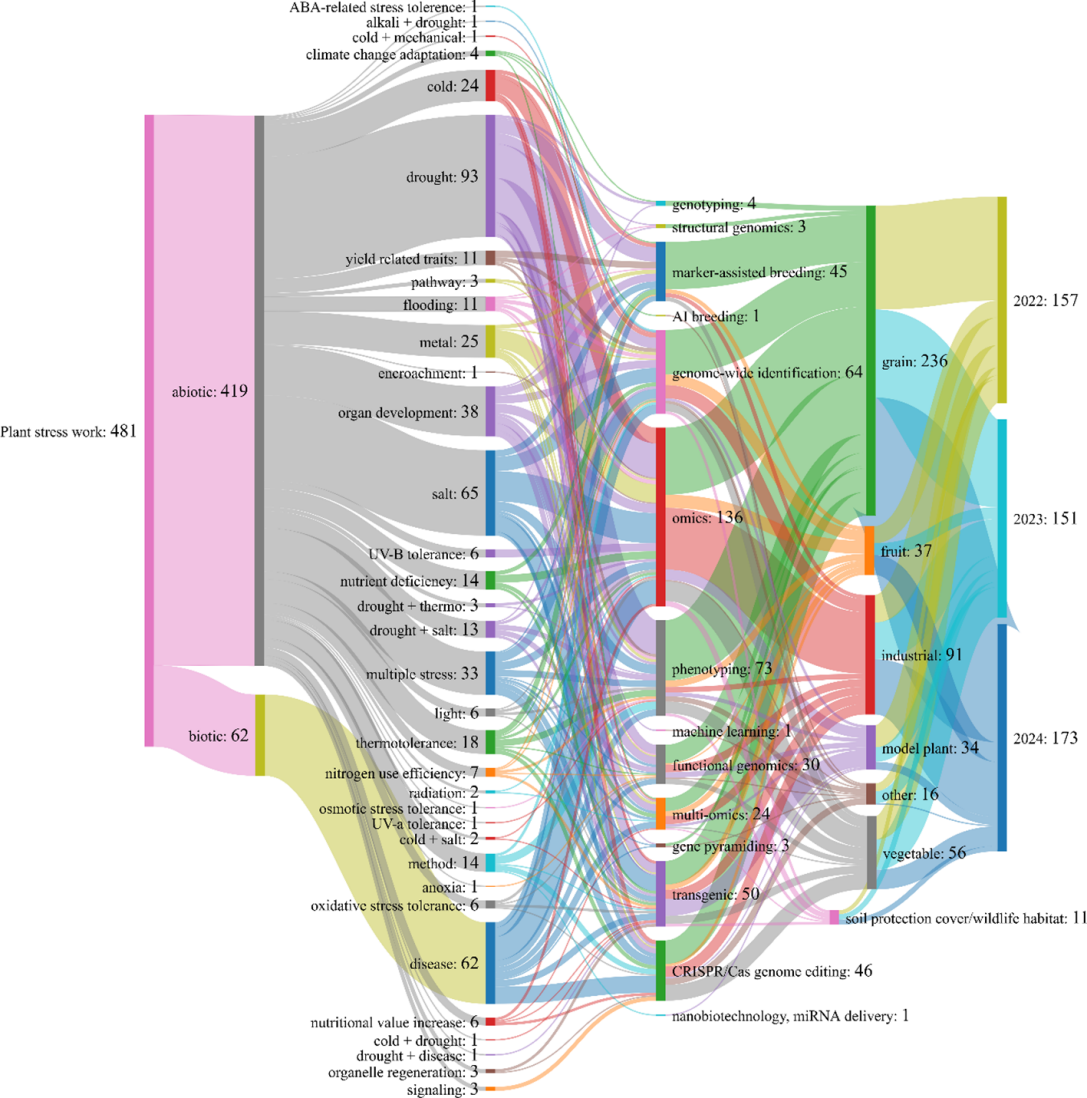
The co-occurrence network of research on plant abiotic and biotic stress was conducted using the latest techniques during the last three years (2022-2024).

Enhancing nutrients and nutrient use efficiency in crops like rice and wheat to reduce fertilizer inputs and environmental impact by optimizing nutrient uptake, translocation, and utilization mechanisms was another avenue in recent works (Kaul et al., 2020). Key areas of progress included the engineering of plant-microbe interactions, the use of endophytes and nanomaterials (nano-biotechnology) (Napier and Sayanova, 2020) to improve growth and stress tolerance, yield and quality traits, and the application of synthetic biology principles to create novel biosynthetic pathways for the production of valuable metabolic compounds like carotenoids, vitamins, and minerals (Sirirungruang et al., 2022; Barnum et al., 2021). The use of biotechnology to improve the nutritional quality of food crops, such as increasing the levels of essential vitamins, minerals, and amino acids, had the potential to address global malnutrition issues (e.g., developing biofortified crops with improved nutritional profiles to address micronutrient deficiencies). In terms of enhanced quality, researchers, for instance, successfully modified oil content and fatty acid amounts in oilseed crops and increased the levels of gamma-aminobutyric acid, a beneficial nutrient, in tomato fruits (Zhu et al., 2020). Editing the *OsLOGL5* gene, which is involved in cytokinin regulation, has led to increased grain yield under multiple environmental conditions. Furthermore, knocking out genes encoding cytokinin oxidase/dehydrogenase enzymes, responsible for cytokinin degradation, resulted in higher yields. Indeed, implementing synthetic biology techniques to modify crops such as *Camelina* and *Arabidopsis* for the improved production of high-value compounds, such as omega-3 acids, plant-derived pharmaceuticals, and renewable chemicals, alongside the development of biofortified crops with better nutritional profiles to combat micronutrient deficiencies (such as elevating the concentrations of vitamins, minerals, and essential amino acids in staple crops like cassava, pearl millet, and sweet potato) represents significant examples of this avenue. Notable examples also include the application of nanoparticles to deliver nutrients and agrochemicals more effectively and the engineering of microorganisms to produce secondary metabolites. Considering all these examples, which generally is directed towards *Arabidopsis*, rice, maize, barley, tobacco, tea, ryegrass, sorghum, rapeseed, alfalfa, cicer, eggplants, wheat, pea, soybean, cotton, faba, cowpea, grapevine, potato, tomato, barley, banana, cabbage, and quinoa, but basal land plants or higher plants such as Marchantia and poplar (**Figure 1**) it is evident that these studies have yielded plants as green factories for producing substances beneficial to humanity, minimizing environmental impact, and enhancing sustainability using GM plants, thereby translating fundamental research into practical products.

## 2 Data collection and visualization methods

The data collection within this study was performed by WOS query (TS = (plant stress$)) AND TS = (“plant biotechnology” OR “agritech” OR “plant genetic engineering” OR “genome editing” OR “CRISPR-Cas” OR “omics*” OR “marker-assisted breeding*” OR “transgenic breeding*” OR “trans-genesis*” OR “cis-genesis*” OR “functional genetic*” OR “targeted modification*” OR “gene overexpression*” OR “targeted genetic engineering*” OR “gene co-overexpression*” OR “gene pyramiding*” OR “phenotyping*” OR “gene mapping*” OR “germplasm evaluation*” OR “climate-smart crops*” OR “tolerant crops”) from the Web of Science Core Collection with publication years refined to 2022, 2023, and 2024. Review articles, books, book sections, conference proceedings, experimental studies, modeling studies, and non-plant and/or stress-irrelevant studies were excluded (records, n=213). During this stage, (after 1118 documents were obtained as sample size), document type was refined as article and Web of Science Categories were refined to plant sciences (records, n=684), agronomy (records, n=144), environmental sciences (records, n=74), (horticulture records, n=49), engineering environmental (records, 23), agricultural engineering (records, n=17), agriculture multidisciplinary (records, n=17), nanoscience nanotechnology (records, n=8), green sustainable science technology (records, n=5) and 912 final documents were obtained in the 2nd round. Then, search results were refined to a more granular level by refining meso and micro-level citation topics, and 644 SCI results were obtained in the 3rd round. A preliminary evaluation was conducted to verify the relevance of the data. Reviews, irrelevant, and duplicate works were also removed from the data set at this stage (excluded irrelevant data were the works analyzing external foliar applications to increase abiotic stress tolerance, halophytes from saline regions, cell wall biosynthesis in yeast, soil culture experiments- basic traditional physiology research- and experiments on virus promoters). A collection of 481 research studies was finally examined following the sequential elections.

The data analysis and visualization were performed for synthesized data set using SigmaPlot Version 13.0. The Sankey diagram was made by https://www.sankeymatic.com/build/while for Word Cloud Chart, an online word cloud generator tool (https://www.jasondavies.com/wordcloud/#http://www.jasondavies.com/wordtree/cat-in-the-hat.txt) was used. The map was created by QGIS mapping software (https://qgis.org).

## 3 Results and discussions

This review analyzed a total of 481 studies on plant stress (**Figure 1**). All of the works were conducted in the last 3 years, with a significant 14.6% increase in the number of studies performed in 2024 compared to 2023, reflecting the growing interest in this critical area. Stress research was classified into two primary categories: abiotic and biotic. Most of the research (87%) focused on abiotic stress, while the remaining portion comprised biotic stress. The study was conducted over 33 separate groups, with drought (22.2%) emerging as the predominant abiotic stressor, followed by salinity (15.5%) and organ development (9%). In biotic stress, only the disease effect was investigated (**Figure 1**). To elucidate the effects of both biotic and abiotic stresses, researchers used 14 different techniques. Although these techniques vary according to the stress factors applied, the omics approach (28.3%) stands out as the most frequently used technique, emphasizing their pivotal role in stress-related research. Other significant techniques included phenotyping (15.2%), genome-wide identification (13.3%), CRISPR/cas genome editing (9.6%), and marker-assisted breeding studies (9.4%). This distribution highlights the growing dependence on integrative, high-throughput techniques to tackle intricate stress systems in plants. On the other hand, it is remarkable that the regulatory constraints and traditional breeding methods leading to lower adaption rate of CRISPR technique (Piergentili et al., 2021).

**Figure 2:**
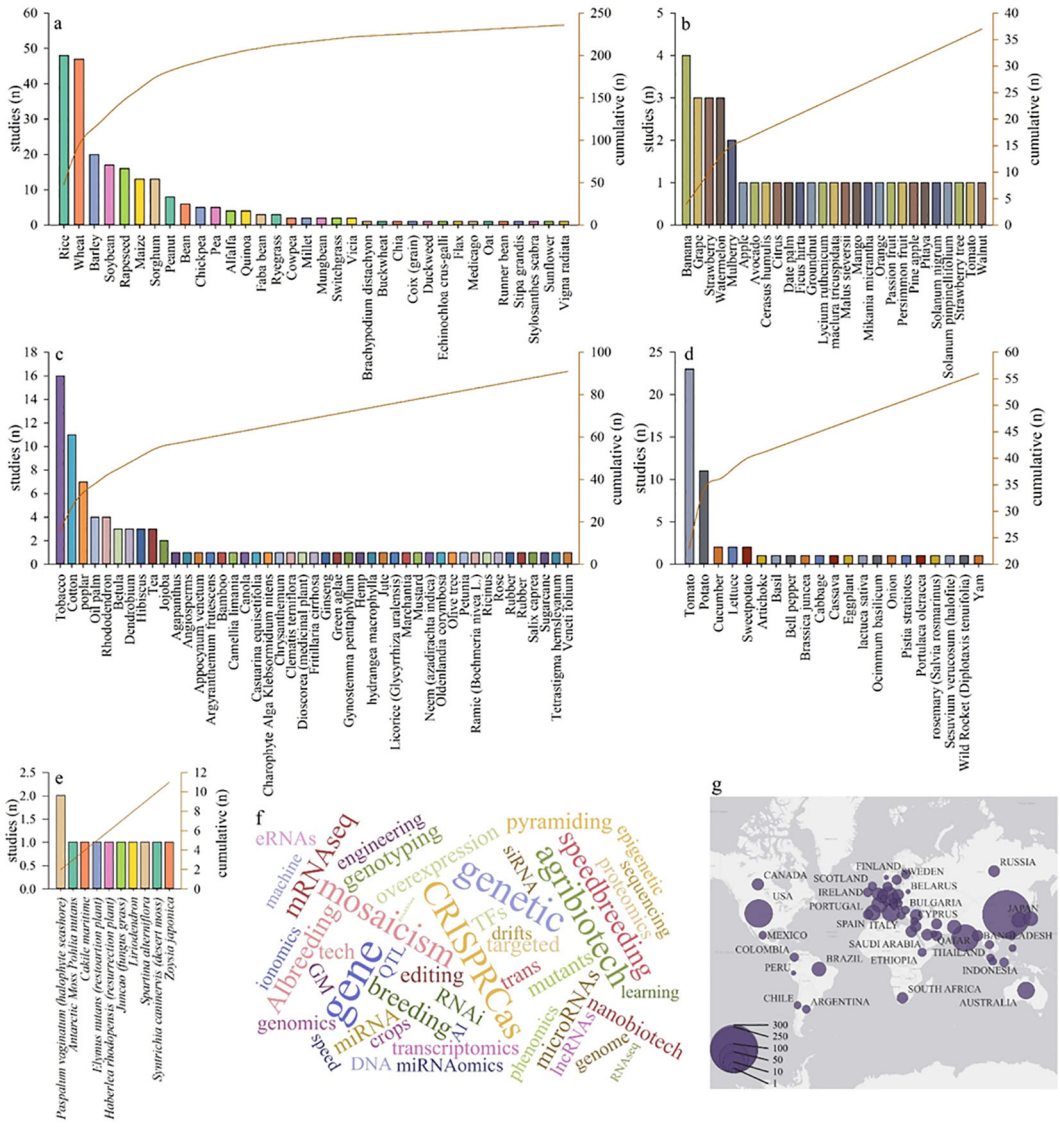
The number of articles by publishing year (The bar chart illustrates published article numbers per year, while the line chart represents the cumulative amount of articles throughout three years). Published articles on grains **(a)** fruits, **(b)** industrial plants, **(c)** vegetables, **(d)** and the plants used as habitat protectors/wildlife soil cover, **(e)**. The word cloud was generated by the keywords in the M&M section **(f)**. Network of contributions among countries based on the national affiliations of all authors **(g)**.

Grains, constituting the dietary staples for an important part of the worldwide population, predominated the studies, accounting for over half of the dataset (49.1%). Industrial crops comprised 18.9%, but vegetables (11.6%) and fruits (7.7%), essential elements of global food security and nutritional diversity, garnered relatively less focus. Among the grains, rice (n=48) and wheat (n=47) were prominent among 34 different species (**Figure 2a**). In the industrial plants, tobacco (n=16) and cotton (n=11) were most prominent (**Figure 2c**). Among the fruits banana being the most remarkable (**Figure 2b**), whereas in the vegetable category, tomato (n=23) and potato (n=11) were the most prevalent (**Figure 2d**). Studies also included plant groups such as habitat protectors or wildlife soil covers, though to a lesser extent (**Figure 2e**). Additionally, a word cloud generated from the most frequent keywords across studies offers insight into trending biotechnological approaches (**Figure 2f**). The research output was geographically dispersed among multiple countries. China emerged as the predominant contributor, generating the highest number of studies, followed by the USA and India (**Figure 2g**). This global participation underscores the acceptance of plant stress research as a priority in addressing the issues posed by climate shifts, food security, and sustainability (**Figure 2g**).

## 4 Prospects of biotechnology for crop improvement

**Figure 3:**
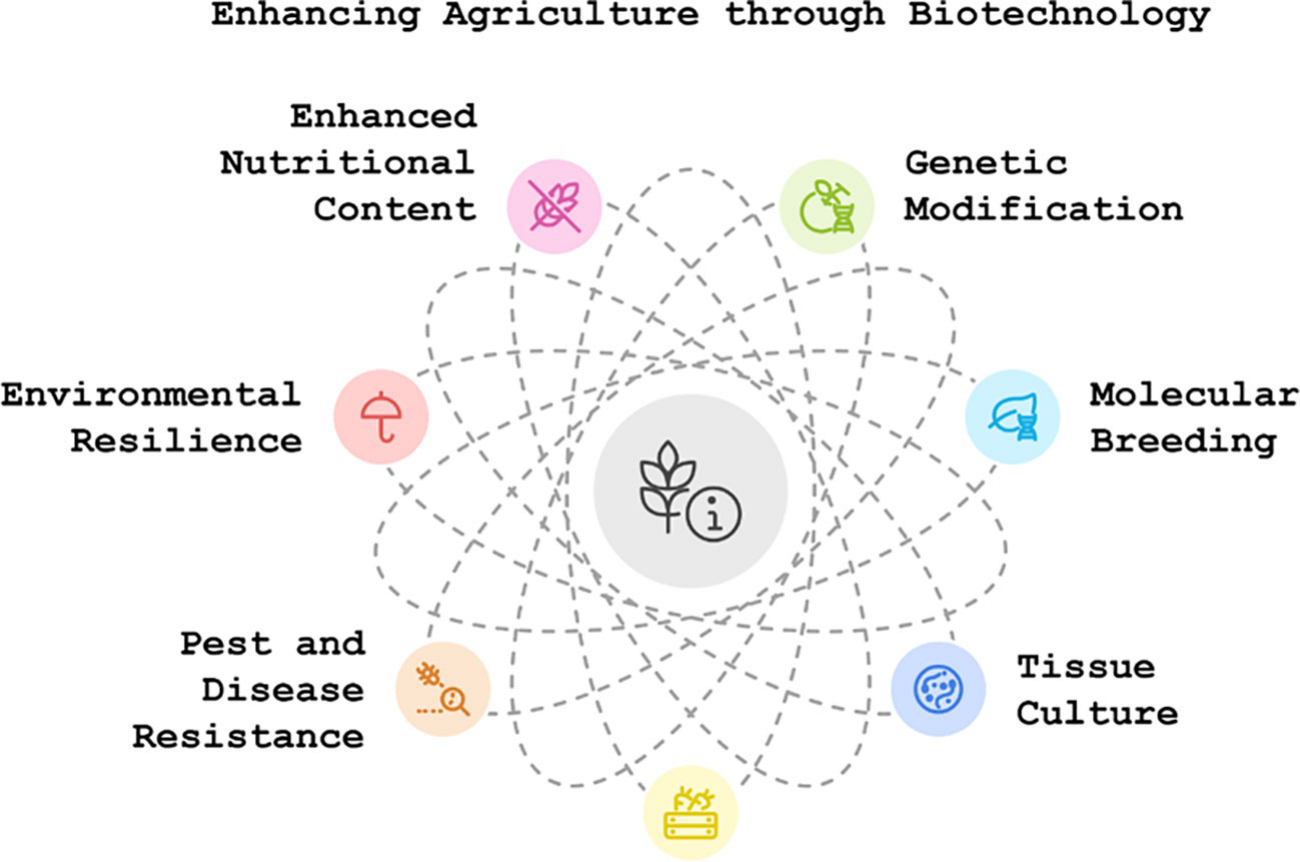
Advancement in agriculture through biotechnological approaches.

The rapid advancement of biotechnology provided plant breeders with access to an extensive array of exceptional genes and characteristics, which can be integrated through singular events into high-yielding and regionally tailored cultivars (**Figure 3**). It presents transformative opportunities by paving the way for engineering plants as “green factories” capable of producing valuable bio-based products, including omega-3 fatty acids, plant-derived pharmaceuticals, and renewable chemicals.

A landmark advancement in the space is CRISPR-Cas technology, offering precise, efficient, and versatility genome editing solution (**Table 1**). Notably, transgene-free gene editing is becoming increasingly viable. Techniques such as using deactivated Cas9 proteins fused with regulatory domains can modulate gene expression epigenetically without altering the DNA sequence (Demirer et al., 2019; Ayanoğlu et al., 2020). Such epigenome-targeted edits have demonstrated potential in controlling developmental traits and stress responses, for instance through manipulation of long non-coding RNAs and enhancer RNAs (Nerkar et al., 2022; Devi et al., 2022).

**Table 1:** Application of CRISPR/Cas system in crops against abiotic and biotic stresses.

Tolerance type	Plant species	Method of delivery	Target gene	Source
Drought tolerance	Rapeseed	Agrobacterium- Mediated	*BnaA6.RGA*	Wu et al. (2020)
	Chickpea	PEG-mediated	*At4CL, AtRVE7*	Badhan et al. (2021)
	Rice	Agrobacterium- Mediated	*OsERA1*	Ogata et al. (2020)
		–	*OsDST*	Santosh Kumar et al. (2020)
		–	*OsPUB67*	Qin et al. (2020)
	Tomato	–	*SlLBD40*	Liu et al. (2020)
	Tomato	–	*SlARF4*	Chen S. et al. (2021)
Salt tolerance	*A.thaliana*	–	*AtWRKY, AtWRKY4*	Li et al. (2021b)
	*A.thaliana*	–	*AtACQOS*	Kim et al. (2021)
	Rice	–	*OsDST*	Santosh Kumar et al. (2020)
		–	*OsNAC45*	Zhang et al. (2020)
		–	*OsAGO2*	Yin et al. (2020)
		–	*OsPQT3*	Alfatih et al. (2020)
		–	*OsPIL14*	Mo et al. (2020)
		–	*OsFLN2*	Chen et al. (2020)
	Tomato	PEG-mediated	*SlHyPRP1*	Tran et al. (2021)
	Wheat	Agrobacterium- Mediated	*TaHAG1*	Zheng et al. (2021)
Heat tolerance	Cotton	Agrobacterium- Mediated	*GhPGF, GhCLA1*	Li et al. (2021a)
	Rice	PEG-mediated	*OsNAC006*	Wang et al. (2020)
	Tomato	Agrobacterium- Mediated	*SlCPK28*	Hu et al. (2021)
Cold tolerance	Rice	–	*OsPIN5b, GS3, OsMYB30*	Zeng et al. (2020)
Metal tolerance	*A. thaliana*	–	*Atoxp1*	Baeg et al. (2021)
	Rice	–	*OsATX1*	Zhang et al. (2021b)
Herbicide resistance	Rice	–	*OsPUT1/2/3*	Lyu et al. (2022)
	Tomato	–	*SlEPSPS*	Yang et al. (2022)
	Tomato	–	*Slpds1*	Yang et al. (2022)

Recent developments in genome editing technologies are transforming plant genetic engineering. Among these innovations, base editing, prime editing, and virus-mediated delivery systems stand out. Base editing, in particular, allows for precise nucleotide changes either transitions or transversions at specific genomic locations without creating double strand breaks (DSBs) or requiring a donor repair template (DRT). Currently, three main types of base editors are utilized: cytosine base editors (CBEs), which convert C:G base pairs to T:A; adenine base editors (ABEs), which change A:T to G:C; and C-to-G base editors (CGBEs), which convert C:G to G:C (Li et al., 2022). This high level of precision makes it possible to induce targeted single-nucleotide changes, leading to either the disruption or enhancement of gene function. As a result, base editing significantly advances gene function analysis, crop improvement, targeted domestication, and the controlled evolution of specific traits in plants (Ren et al., 2018; Tan et al., 2022; Li et al., 2022). Prime editing is an advanced and precise genome editing method that enables targeted DNA modifications without requiring double-strand breaks or donor DNA, thereby minimizing off-target effects (Averina et al., 2024; Anzalone et al., 2019, 2020; Gao, 2021). It uses a Cas9 nickase fused to a reverse transcriptase and a specialized guide RNA (pegRNA) to introduce desired edits through reverse transcription and DNA repair mechanisms (Xie et al., 2022). Prime editing was first demonstrated in plants in 2020 using rice and wheat protoplasts, showing its potential for precise genome modification (Lin et al., 2020). Optimized plant-specific systems enabled targeted point mutations, insertions, and deletions, with editing efficiencies reaching up to 21.8%. Subsequent studies established stable rice lines with desired edits in both endogenous and exogenous genes (Li et al., 2020; Tang et al., 2020). Notably, prime editing has been used to modify key agronomic genes like ALS, IPA, and TB1, enhancing traits such as herbicide resistance and yield-related architecture (Butt et al., 2020). Additionally, virus-based delivery systems, which facilitate efficient and heritable genome edits in plants, overcoming traditional tissue culture limitations (Zhang et al., 2022). To increase editing efficiencies, Weiss et al., 2025 achieved an advantageous miniature system/transgene-free editing of Arabidopsis thaliana in a single step with edits inherited in the next generations by engineering tobacco rattle virus to carry a compact RNA-guided enzyme and its guide RNA. They showed that even though viruses might have low cargo capacities to carry big CRISPR-Cas systems for targeted mutagenesis (e.g., meganucleases) (Honig et al., 2015), using viral delivery as vectors to introduce genome editing reagents to plants is a novel platform.

## 5 Sustainable agriculture

Protecting the environment is the primary goal of sustainable practices because food security goes hand-in-hand with sustainable agriculture. Therefore, practices that conserve resources and protect ecosystems are essential. Critical aspects of sustainable agriculture include the multifunctional platform of synthetic plant biology, like exogenous application of RNA interference molecules (RNAi) or gene editing, which can potentially address the challenge of feeding a rapidly growing global population (Demirer et al., 2019). Additionally, bioinformatic tools that enabled the identification of conserved motifs and sequence similarities between organisms have contributed significantly to the development of more efficient, productive, and environment-friendly agriculture (Zhu et al., 2020). Genetically modified (GM) crops have significantly revolutionized agriculture and food security, particularly for grains such as maize, rice, and soybean, as well as industrial crops such as cotton, tea, and tobacco. These crops have shown enhanced traits like heavy metal tolerance, drought tolerance, pest and disease resistance, herbicide tolerance, and improved food quality, farm income, and environmental benefits. Some well-known examples that have already reached a plateau serving precision agriculture include plants expressing Bt toxins and virus-resistant papaya, squash, and plum (Kholová et al., 2021; Ricroch et al., 2017; Sharma et al., 2002; Liu et al., 2017). Despite these advancements, the applications of GM technologies have been confined mainly to commodity crops, leaving minor crops, fruits, and vegetables underrepresented. This is concerning, as these underutilized crops are essential for a nutritionally balanced diet and for diversifying agricultural income sources (Cao et al., 2016). Expanding the scope of biotechnological research to include these crops can address regional food security challenges and increase the robustness of global food systems.

Conventional plant genetic modification methods has been reported unfeasible for many economically important crops, such as common bean (Weiss et al., 2025). Even CRISPR today remains sometimes ineffective in rice (particularly Japonica cultivars) (Shan et al., 2014) and wheat (the hexaploid nature of wheat, which has multiple sets of chromosomes, complicates CRISPR applications) (Zhang et al., 2021a). Some potato and tomato varieties also pose regeneration challenges, making it difficult to implement CRISPR-based edits in a large-scale, commercially viable manner. Nonetheless, the advent of CRISPR/Cas9 has paved a new way to rapidly modify a broader range of plants, including fruits, vegetables, and orphan crops that are understudied/underrepresented but critical for regional food security (Zhu et al., 2020) in one step and only one generation (Weiss et al., 2025). These developments have the potential to transform agriculture and make it more regenerative, productive, and resilient in the face of global fluctuations like climate change, limited land and water resources, and the need to feed a significant population (Saeed et al., 2023).

## 6 Food security

Biotechnology-driven supply systems have changed the game for food security by developing crop varieties with higher yields, better disease resistance, and better biotic and abiotic stress tolerance. These advancements are crucial for 700 million people worldwide who are living on less than $1.90 daily and suffering from malnutrition (World Extreme Poverty Statistics, 2024). State-of-the-art technology trends can contribute to a significant increase in the desirable regulated products characterized by high, medium, and very high product purity, especially in cereal, fruit, and vegetable crops (Zhu et al., 2020). Deleting unfavorable genetic elements or introducing gain of function mutations to create perfect germplasms efficiently to establish male sterility (Singh et al., 2018), or formation of haploid embryos (Liu et al., 2017), or domestication (Li et al., 2018). These advancements make agriculture more environmentally friendly and economical. They also may conserve local varieties protect crop production with less labor, providing a sustainable food supply for a growing global population. However, food security is interconnected with many other global challenges (Cole et al., 2018), and addressing it requires a multifaceted approach that covers environmental, social, and economic factors (Calicioglu et al., 2019). For instance, the scientific dominance in stress research by some countries could be characterized by a combination of environmental, economic, and technological drivers. The countries with high climatic stressors, e.g., long periods of drought, extreme temperatures, or soil salinity, experience higher pressures to develop stress-resistant crops. Their research agenda might be, therefore, a reflection of the urgency to provide food security and sustain agricultural production under extremely unpredictable weather conditions. Moreover, countries with strong agriculture economies, particularly those that rely mainly on staple crops, might also be large investors in stress tolerance (Li et al., 2018). Their governments and research institutions may know the strategic importance of stable yields, not only to ensure national food security but also to feed export markets and industrial applications such as biofuels and animal feed. In such cases, scientific investment is a mix of economic pragmatism and necessity (Cole et al., 2018). Apart from economic and environmental factors, the technological and infrastructural capacity of a country also decides research output. Countries with advanced research infrastructures and well-funded and established industries are better placed to conduct cutting-edge research on plant stress tolerance. Also, fostering collaborative efforts through grants/projects on climate adaptation and strengthening crop value chains and knowledge sharing have been cornerstones (Calicioglu et al., 2019). Because consumption through the sharing of knowledge products might encourage the exchange of best practices and experiences among countries to enhance crop production and consumption. These factors being responsible for the dominance of some countries in stress research bring into question a pertinent issue of research equity at the global level.

Since agriculture is the backbone of many economies, improved food security strengthens the agribiotech sector, creating new jobs and boosting incomes (Prado et al., 2014). Reliable food production also helps stabilize food prices, benefiting consumers and producers and giving rise to market stability. Moreover, food security isn’t just about quantity but also quality (Varzakas and Smaoui, 2024). Therefore, access to nutritious food can help combat diet-related diseases and proper nutrition and strengthen immune systems, making populations more resilient to diseases and/or malnutrition.

## 7 Climate change resilience

Climate change significantly threatens world food production by increasing its frequency (Gupta et al., 2020); therefore, obtaining plasticity with novel abilities for plants is essential in light of the increasing effects of climate change. Agricultural practices contribute to climate change; however, they can also be a part of the solution to climate change mitigation. Indeed, conventional and novel plant breeding efforts have produced crop varieties with improved tolerance to drought, heat, cold, soil salinity, etc., all of which are intensified by climate change. The innovations enhanced stress tolerance in crops, thereby ensuring sustained productivity and yield stability in the face of shifting environmental conditions (Zhu et al., 2020; Ricroch et al., 2017; Cao et al., 2016; Demirci et al., 2017; Koltun et al., 2018). This advancement is particularly crucial for major staple grains such as maize, wheat, and rice, which form the dietary foundation for the majority of the global population. In accordance with this, cereal growth should be prioritized to be protected from abiotic/biotic stress by countries with high dependence on staple foods. Moreover, grains occupy the greatest area under cultivation in global agricultural production, hence there has been a concentration of research investment on these crops (most of the countries undertake large-scale projects specifically to sustain cereal production, and these are typically supported by significant funding). These plants also possess extremely high economic significance, not only as food but also for biofuel, animal feed, and industrial purposes. Apart from this, plants such as rice and maize are experimental crops in genetic studies and are therefore, always under the microscope. Cumulatively, all these factors result in more research on grains as compared to other plant groups. In addition, the distribution of funds for vegetables and fruits frequently relies on regional priorities, particular health and nutrition objectives, and the strategic interests of commercial organizations. However, fruits and vegetables are equally important to human nutrition, and therefore research must be carried out on both as well.

The integration of nanomaterials in agriculture, on the other hand, offers a transformative strategy to enhance nutrient delivery and agrochemical efficiency (Usman et al., 2020). Key benefits include reduced fertilizer waste, optimized nutrient utilization, improved crop yield and quality (Quintarelli et al., 2024), and activation of plant defenses against stresses such as drought and salinity (Zulfiqar et al., 2024). Nonetheless, concerns about environmental toxicity (Zulfiqar et al., 2024) remain among critical challenges. Addressing these issues is crucial to comprehending and harnessing nanotechnology’s potential for climate resilience.

## 8 Regulatory frameworks

Regulatory frameworks must strike a balance between rigorous safety assessments and efficient approval processes to enable the timely delivery of innovations that can enhance food security and climate change resilience. A flexible, evidence-based, and transparent regulatory framework has been crucial for the smart advancement and use of agricultural biotechnologies to date (Hood et al., 2011; Raman, 2017). Today, robust and adequate regulatory frameworks that can effectively assess and manage the safety and environmental impact of biotechnology-derived crops are more than needed, given the risks diminishing natural resources. However, while regulatory approval is essential for commercialization, this can also pose significant hurdles and delays, hindering the timely delivery of beneficial products to farmers and consumers (Lassoued et al., 2018). This regulatory uncertainty can create barriers to the widespread implementation of advanced techniques that have the potential to increase crop productivity, climate resilience, and safe farming practices (Lassoued et al., 2018). Streamlining and harmonizing regulations internationally can help accelerate the translation of research advances into practical applications (Entine et al., 2021).

## 9 Public perception and trust

Public views and attitudes toward emerging biological sciences and novel technologies are critical determinants of the successful launch and implementation of these technologies (Siegrist and Hartmann, 2020); however, there are questions as to whether they are currently sufficiently taken into account which may end up leading ‘market avoidance’. Indeed, failure to address public concerns and maintain trust may have a negative impact on novel techniques and their applications (Trump et al., 2023). Public safety concerns about the environmental impact/ecological balance and ethical standards of agribiotech, therefore, must be addressed by fostering an environment of transparency, open communication, and inclusive engagement with all stakeholders, including the general public, farmers, policymakers, and industry (Trump et al., 2023). Promoting public awareness and establishing clear and robust regulatory frameworks, as well as effective communication strategies, can help navigate the latest advancements in the area and ensure the responsible development and deployment of agritech technologies in a digital era (Kleter et al., 2019).

## 10 Risks and benefits

The potential of novel plant genetic modification methods is vast, as it spans the full spectrum of life, including agriculture, food processing, medicine, and various other fields. Indeed, the beneficial outcomes and processes for advancement lead to many life-saving agents, like vaccines, proteins, etc., that have been developed lately (Demirer et al., 2019). Yet, the applications is not without risks and limitations. Despite several advantages, they are consistently seen as posing a potential risk to both the environment and people’s health in various respects (Zhu et al., 2020). In fact, this tremendous progress in the area has limited the majority of resources (Zhang et al., 2019). Therefore, the intentional alteration and transmission of precisely engineered gene constructs through modern methodologies have had generated ongoing global controversy (Demirer et al., 2019). Now, these technologies may not go beyond if ethical issues are clearly articulated, including informed consent, confidentiality, research subjects, equitable access to research benefits, mortality, and benefits for research personnel such as insurance, intellectual property rights, and liability (Prado et al., 2014). Hence, considerable attention should be directed to the management of bioethics/bioterrorism through the utilization of gene-edited super pathogens or pharmaceuticals designed to eliminate memory and immunological responses.

## 11 Socioeconomic implications

The rapid development of genetic modification tools has brought about significant socioeconomic implications, particularly in the domain of the agri-food sector. For example, after the creation of GABA-enriched tomatoes in 2022 (Waltz, 2022), the first CRISPR-edited vegetable (mustard) hit the US market as a start-up last year. Editing a healthier future with these techniques is encouraging, as evident in instances such as removing allergens from foods (Assou et al., 2022); however, the extensive use of these technologies has elicited apprehensions regarding the possible consolidation of power among a limited number of large corporations potentially exacerbating economic disparities and limiting the access of small-scale farmers to these innovations (Braun, 2002; Trump et al., 2023). Additionally, the patenting of the products and the rights associated with them have sparked debates around equitable access and the implications for food security, maybe not for developed but in developing countries (Munshi and Sharma, 2017). Another socioeconomic implication is the potential impact on trade and global markets, as countries and continents/regions have adopted different regulatory approaches to the use of these products, leading to trade barriers and disruptions (Trump et al., 2023). Addressing these socioeconomic concerns requires a balanced and inclusive approach, ensuring that the benefits are equitably distributed and that the needs of diverse stakeholders, including small-scale farmers, consumers, and vulnerable communities, are taken into account (Braun, 2002; Munshi and Sharma, 2017).

## 12 Ethical considerations

CRISPR-edited crops are positioned outside the regulatory restrictions typically applied to GMOs, offering a more flexible framework for their development/adoption (Ding et al., 2022; Pan et al., 2021). Nevertheless, this strategy in agriculture might still not universally acceptable due to the variances in ethical, cultural, and regulatory views of countries. In France and the European Union, for instance there are strict regulations due to public caution and environmental concerns (Gaskell et al., 2010). In addition, in Latin America, nations such as Mexico and Ecuador reject gene-edited crops to maintain biodiversity and local heritage (Montenegro de Wit, 2017). Also, distrust in Africa and Asia, Tanzania and India, is based on seed sovereignty as well as health and environmental impacts (Paarlberg, 2010; Jasanoff and Hurlbut, 2018). Furthermore, religious and cultural beliefs are also reflected in public views, with some groups viewing gene editing as unnatural or immoral, even if it is not directly illegal.

On the other hand, potential misuse (e.g., bioterrorism, genetic mosaicism, non-target gene drifts), such as introducing allergens, invasive pest species, or viruses, leads to ecological imbalance as in the case of other ethical considerations surrounding germline editing, as well as in breeding of the human species (eugenics) (Ayanoğlu et al., 2020) are among the critical issues in emerging agricultural technologies developed for the safety of food stocks. A balanced approach is needed to maximize benefits while mitigating risks and ensuring responsible innovation by both life scientists and social scientists. Overlooking to address the product’s legal and social implications can create substantial obstacles to product acceptance and adoption, hence supporting the implementation of the “safety-by-design” supremacy ideology (Annas et al., 2021). Therefore, legal and social challenges and opportunities are to be considered for sustainable and responsible agri-biotech applications.

## 13 Conclusion

Obtained data reveals key trends and gaps in abiotic stress research in crops. In this context, we concluded that while omics-based studies dominate the field, CRISPR/Cas genome editing is gaining traction as an emerging tool. The focus remains largely on grain crops, with fruits and vegetables receiving considerably less attention. Among species, *Oryza sativa* has been a primary model for improving stress tolerance in recent years. Drought stress is the most extensively studied factor, particularly in grains, whereas research on other abiotic stresses, remains scarce. These findings underscore the need for a more balanced research focus across different crop types and stress factors to ensure comprehensive strategies for improving crop resilience. On the other hand, the rapid advancements in biotechnology for crop improvement have brought both significant benefits and pressing socioeconomic and ethical concerns that require careful consideration. While cutting-edge technology has enabled enhanced yields, resilience, and reduced reliance on chemical inputs, issues such as unequal access to technology, potential monopolization by large corporations, trade barriers and varying levels of public acceptance across regions present substantial barriers to equitable implementation.

In some countries or cultures, the use of genome editing or GM-based approaches may face ethical, legal, or religious objections, which can influence regulatory pathways and societal acceptance. Addressing these concerns through inclusive policymaking, transparent communication, and public engagement is critical to ensure trust and responsible innovation.

Future research priorities in this context, may cover increasing representation of underrepresented/underutilised crops and need for integrative and interdisciplinary methods. Ultimately, the successful and responsible application of agricultural biotechnology hinges on maintaining technological integrity, ensuring safety and security, and transparently conveying the legal, ethical, and social implications of these transformative tools. By proactively addressing the key challenges, the biotechnology community can foster sustainable innovations that serve the diverse needs of stakeholders, from small-scale farmers to consumers and vulnerable communities.
